# Dysbiosis of the Salivary Microbiome Is Associated With Non-smoking Female Lung Cancer and Correlated With Immunocytochemistry Markers

**DOI:** 10.3389/fonc.2018.00520

**Published:** 2018-11-20

**Authors:** Junjie Yang, Xiaofeng Mu, Ye Wang, Dequan Zhu, Jiaming Zhang, Cheng Liang, Bin Chen, Jingwen Wang, Changying Zhao, Zhiwen Zuo, Xueyuan Heng, Chunling Zhang, Lei Zhang

**Affiliations:** ^1^College of Life Science, Shandong Normal University, Jinan, China; ^2^College of Life Science, Qilu Normal University, Jinan, China; ^3^Clinical Laboratory and Core Research Laboratory, The Affiliated Central Hospital of Qingdao University, Qingdao, China; ^4^Qingdao Human Microbiome Center, The Affiliated Central Hospital of Qingdao University, Qingdao, China; ^5^Qingdao Institute of Oncology, The Affiliated Central Hospital of Qingdao University, Qingdao, China; ^6^Microbiological Laboratory, Department of Infection Management, Department of Neurosurgery, Lin Yi People's Hospital, Linyi, China; ^7^School of Information Science and Engineering, Shandong Normal University, Jinan, China; ^8^Shandong Children's Microbiome Center, Qilu Children's Hospital of Shandong University, Jinan, China; ^9^Beijing Advanced Innovation Center for Big Data-Based Precision Medicine, School of Chemistry and Environment, Beihang University, Beijing, China; ^10^Department of Respiratory Medicine, The Affiliated Central Hospital of Qingdao University, Qingdao, China; ^11^Shandong Institutes for Food and Drug Control, Jinan, China

**Keywords:** salivary microbiome, dysbiosis, biomarker, non-smoking female patient, lung cancer

## Abstract

**Background:** Association between oral bacteria and increased risk of lung cancer have been reported in several previous studies, however, the potential association between salivary microbiome and lung cancer in non-smoking women have not been evaluated. There is also no report on the relationship between immunocytochemistry markers and salivary microbiota.

**Method:** In this study, we assessed the salivary microbiome of 75 non-smoking female lung cancer patients and 172 matched healthy individuals using 16S rRNA gene amplicon sequencing. We also calculated the Spearman's rank correlation coefficient between salivary microbiota and three immunohistochemical markers (TTF-1, Napsin A and CK7).

**Result:** We analyzed the salivary microbiota of 247 subjects and found that non-smoking female lung cancer patients exhibited oral microbial dysbiosis. There was significantly lower microbial diversity and richness in lung cancer patients when compared to the control group (Shannon index, *P* < 0.01; Ace index, *P* < 0.0001). Based on the analysis of similarities, the composition of the microbiota in lung cancer patients also differed from that of the control group (r = 0.454, *P* < 0.001, unweighted UniFrac; r = 0.113, *P* < 0.01, weighted UniFrac). The bacterial genera *Sphingomonas* (*P* < 0.05) and *Blastomonas* (*P* < 0.0001) were relatively higher in non-smoking female lung cancer patients, whereas *Acinetobacter* (*P* < 0.001) and *Streptococcus* (*P* < 0.01) were higher in controls. Based on Spearman's correlation analysis, a significantly positive correlation can be observed between CK7 and Enterobacteriaceae (r = 0.223, *P* < 0.05). At the same time, Napsin A was positively associated with genera *Blastomonas* (r = 0.251, *P* < 0.05). TTF-1 exhibited a significantly positive correlation with Enterobacteriaceae (r = 0.262, *P* < 0.05). Functional analysis from inferred metagenomes indicated that oral microbiome in non-smoking female lung cancer patients were related to cancer pathways, p53 signaling pathway, apoptosis and tuberculosis.

**Conclusions:** The study identified distinct salivary microbiome profiles in non-smoking female lung cancer patients, revealed potential correlations between salivary microbiome and immunocytochemistry markers used in clinical diagnostics, and provided proof that salivary microbiota can be an informative source for discovering non-invasive lung cancer biomarkers.

## Introduction

Lung cancer is considered the leading cause of cancer death worldwide, accounting for over 300,000 deaths annually (1). Although lung cancer is often recognized as a disease suffered by smokers, global statistics estimate that 15% of male lung cancer and 53% of female lung cancer are not attributable to tobacco use (2, 3). Non-smoking lung cancer patients are often considered a different population (4). Non-small cell lung cancer (NSCLC) in non-smokers is clinically characterized by a higher occurrence of adenocarcinoma and an increased incidence in females, when compared to NSCLC in smokers (5).

Recently, the study of oral microbiome has developed from oral diseases to systemic diseases, even systemic cancers (6–12). It has been proposed that oral microbiome play a causal role in the dynamic equilibrium with the immune-inflammatory response of the host (13, 14). The human respiratory tract is the primary and consistent entry point for numerous microorganisms, primarily airborne, but also those transferred through saliva. Oral bacterial communities may originate from the oropharynx and tracheobronchial or from the environment through inhalation, and probably seed the lungs with oral bacteria (15, 16). The association extends into the systemic circulation and holds the answer for understanding disease and developing non-invasive approaches to health care (17–20). However, cigarette smoking may lead to oral microbiome imbalance, thereby causing potential shifts in functional pathways, and having implications for smoking-related diseases (21). Previous studies on oral microbiome in lung cancer have not stratified clinical samples based on the smoking status and fully evaluated confounding factors such as smoking on the discovered bacterial biomarkers (18). Furthermore, there has been no report on characterizing oral microbiome in non-smoking female lung cancer patients.

It is considered that environmental factors and genetic susceptibility may contribute to risk of lung cancer in non-smokers (22, 23). The main goal of this paper is to place into perspective different experimental and methodological views, to better understand the effect of oral microbiota changes on disease onset or during different disease stages. In this pilot study, we performed a comprehensive comparison of the salivary microbiota of non-smoking female lung cancer patients and that of healthy control subjects, using 16S rRNA gene sequencing. We characterized the variation in salivary microbiome balance in non-smoking female lung cancer patients and the dysbiosis of the salivary microbiome, based on structure, composition, and function. We also identified the relationship between salivary microbiota and immunocytochemistry markers containing thyroid transcription factor(TTF-1), Napsin A and cytokeratin(CK7), as well as the specific microbial signatures of lung cancer (24, 25). The role of oral microbiota composition is important to evaluate how salivary microbial biomarkers at the community level could improve assessment for individuals and populations at risk, especially with respect to developing non-invasive diagnostic tests.

## Methods

### Study design and cohort information

This study was conducted as per the recommendations of Human Specimen Study guidelines of the Institutional Review Board of the Affiliated Central Hospital of Qingdao University (IRB# QCH16-1101-01). The study was designed based on the principle of PRoBE design (prospective specimen collection before outcome ascertainment and retrospective blinded evaluation) (26). The critical feature of PRoBE design involves prospective clinical sample collection from a study cohort relevant to the clinical application, prior to ascertaining the outcome. Biomarker tests intended for FDA approval and clinical use must incorporate PRoBE principles at an early stage, as these principles eliminate potential biases commonly seen at the discovery stage (26).

Newly diagnosed and untreated non-smoking female lung cancer patients and matched healthy controls were recruited for this study from the Affiliated Central Hospital of Qingdao University. The inclusion criteria for the patients required that they be female and have confirmed diagnosis of non-small-cell lung cancer. Exclusion criteria included a history of smoking or drinking, evidence of locally advanced lung cancer, metastatic lung cancer, chemotherapy, or radiation therapy prior to saliva collection and diagnosis of other malignancies within 5 years from the time of saliva collection. Healthy control individuals were matched based on age, gender, smoking and drinking status.

Written informed consents and questionnaire data sheets were obtained from all participants who agreed to act as sample donors, in compliance with national legislation and the Code of Ethical Principles for Medical Research Involving Human Subjects of the World Medical Association (Declaration of Helsinki). All methods and experimental protocols in this study were performed in accordance with appropriate guidelines and standard operating procedures.

### Sample collection, process and storage

All subjects were requested to refrain from drinking, eating and using oral hygiene products for at least 2 h prior to sample collection. Unstimulated whole saliva was consistently collected, processed, and stored according to previously established protocol (27, 28). Briefly, 5~10 mL of unstimulated whole saliva was collected in a 50 mL sterile tube from each subject from 9 to 10 a.m. (the collection time was <30 min and the collection tubes were kept on ice after collection). Whole saliva samples were then centrifuged at 2,600 × g for 15 min at 4°C. Following this, the supernatant was carefully removed and the pellet was immediately frozen and stored at −80°C prior to assay. The immunocytochemistry analysis of the resected tumors was performed using 5-μm thick, formalin-fixed, paraffin-embedded tissue sections of each case, and a BenchMark Autostainer with the EnVision detection system was used to stain all slides (29, 30). Clinical characteristics of samples and results of immunocytochemistry analysis are shown in Table S1.

### DNA extraction and 16S rRNA gene sequencing

The DNA was extracted using the UltraClean Microbial DNA Isolation Kit (MO BIO Laboratories Inc, Carlsbad, California, USA) as per the manufacturer's instructions. The equivalent of 1 ul of each sample was then used for DNA quantification using a NanoDrop 2000 Spectrophotometer (Thermo Scientific). The hypervariable region V1-V2 of the 16S rRNA gene was amplified to analyze the bacterial populations in the samples. 16S ribosomal RNA (or 16S rRNA) is the component of the 30S small subunit of a prokaryotic ribosome that binds to the Shine-Dalgarno sequence. The gene coding for it is referred to as 16S rRNA gene and it is used in reconstructing phylogenies, due to the slow rates of evolution of this region of the gene (31, 32).

PCR was conducted using the bacterial universal primers F27 (5′-AGAGTTTGATCMTGGCTCAG-3′) and R338 (5′-GCTGCCTCCCGTAGGAGT-3′). A QIAquick PCR Purification Kit (Qiagen, Barcelona, Spain) was used to initially purify the amplicons, which were then quantified using a NanoDrop 2,000 Spectrophotometer (Thermo Scientific) and then pooled in equal concentrations. Illumina HiSeq 2,500 next-generation sequencer (Illumina Inc., San Diego, CA, USA) was then used to sequence the pooled amplicons (2 nM), following standard Illumina platform protocols. All sequencing data of this study was then uploaded to the NCBI SRA database (accession number: SRP145042). The SRA database webpage is https://www.ncbi.nlm.nih.gov/sra/.

### Taxonomy quantification using 16S rRNA gene sequences and statistical methods

As described by Magoč and Salzberg (33), the raw FASTQ files were first de-multiplexed, then quality-filtered using Trimmomatic and merged using FLASH with the following criteria: (i) All reads at any site receiving an average quality score <20 over a 50-bp sliding window, were truncated; (ii) The primers were exactly matched allowing 2 nucleotide mismatching, and reads with ambiguous bases were removed. (iii) Sequences whose overlaps were longer than 10 bp were merged according to the overlapping sequence.

All sequence analyses were conducted in the Quantitative Insights Into Microbial Ecology (QIIME, version 1.9.1) software suite (34), according to the QIIME tutorial (http://qiime.org/). Usearch61 was used with *de novo* models to remove chimeric sequences (35). Sequences were then clustered against the 2013 Greengenes (13_8 release) ribosomal database (97% reference data set). Sequences not matching any of the entries in this reference were subsequently clustered into *de novo* operational taxonomic units (OTUs) at 97% similarity with UCLUST. The RDP classifier within QIIME and the Greengenes reference data set were used to assign taxonomy to all OTUs (36). Alpha diversity and rank abundance scripts within the QIIME pipeline were used to calculate rarefaction and rank abundance curves from OTU tables. Unweighted pair group method with arithmetic mean (UPGMA) clustering (also known as average linkage) on the distance matrix of OTU abundance was used to perform hierarchical clustering based on the population profiles of the most common and abundant taxa. The QIIME package was then used to obtain a newick-formatted tree.

The potential bacterial biomarkers were explored using linear discriminant effect size (LEfSe)—an algorithm for high-dimensional biomarker discovery that uses linear discriminant analysis (LDA) to estimate the effect size of each taxon that is differentially represented in cases and controls. In addition to detecting significant features, LEfSe also ranks features by effect size, and places features explaining most of the biological difference at the top (37). The least number of sequences present in any given sample from a sample category were randomly selected prior to calculating community-wide dissimilarity measures (*alpha* diversity and *beta* diversity), to account for any bias caused by uneven sequencing depth. The OTU table was then rarefied to a sequencing depth of 11,000 per sample for both diversity analyses. All principal coordinate analyses (PCoA) were based on unweighted UniFrac distances using evenly sampled OTU abundances. The prediction of the functional composition of a metagenome with marker gene data and a database of reference genomes was performed using phylogenetic investigation of communities, by reconstructing the unobserved states (PICRUSt, Version 1.1.1), as described by Langille et al (38).

Graphical representations of the results were created using STAMP (39). Unless otherwise stated, the data are presented as Mean ± SD. Continuous variables between independent samples were compared using the Mann-Whitney test or the unpaired-sample *t*-tests. Results with *P*-values <0.05 were considered statistically significant. The Spearman's rank correlation was used to determine the statistical dependence between continuous variables. Specifically, the analyses were performed with the ANOSIM test for differences in microbial community composition. The SPSS statistical package, version 24.0 (SPSS) was used to perform all analyses.

## Results

### Participants

In all, 247 participants including 75 non-smoking female lung cancer patients and 172 healthy controls were recruited for this study. The lung cancer group and control group were matched for age (61.50 ± 9.32 vs. 59.04 ± 7.79, respectively, *P* = 0.104).

Demographics and clinical profiles of all subjects (75 cancer patients and 172 healthy controls) are presented in Table S1.

### Decrease in microbial diversity and richness in non-smoking female lung cancer patients

**Figure 1:**
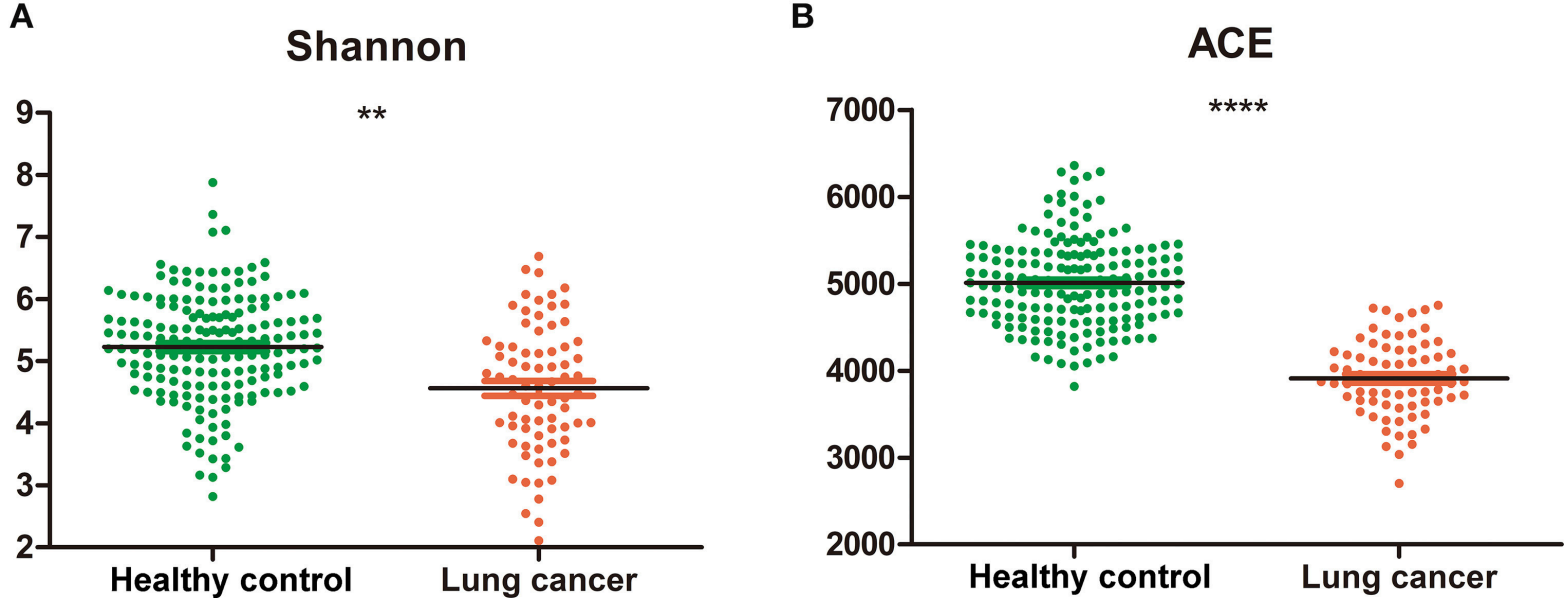
Diversity and richness of oral microbiota in cancer and control. **(A)** Shannon index; **(B)** Ace. **P < 0.01, ****P < 0.0001, unpaired t-test.

The alpha diversity of the salivary microbiota of non-smoking female lung cancer patients was lower than that of healthy controls, in terms of both diversity (Shannon index, 4.56 ± 1.03 vs. 5.22 ± 0.85, *P* < 0.01, Figure 1A) and richness (Ace index, 3911.04 ± 432.36 vs. 5009.81 ± 509.10, *P* < 0.0001, Figure 1B).

### Relative taxon abundance in microbiota of lung cancer patients and control subjects

**Figure 2:**
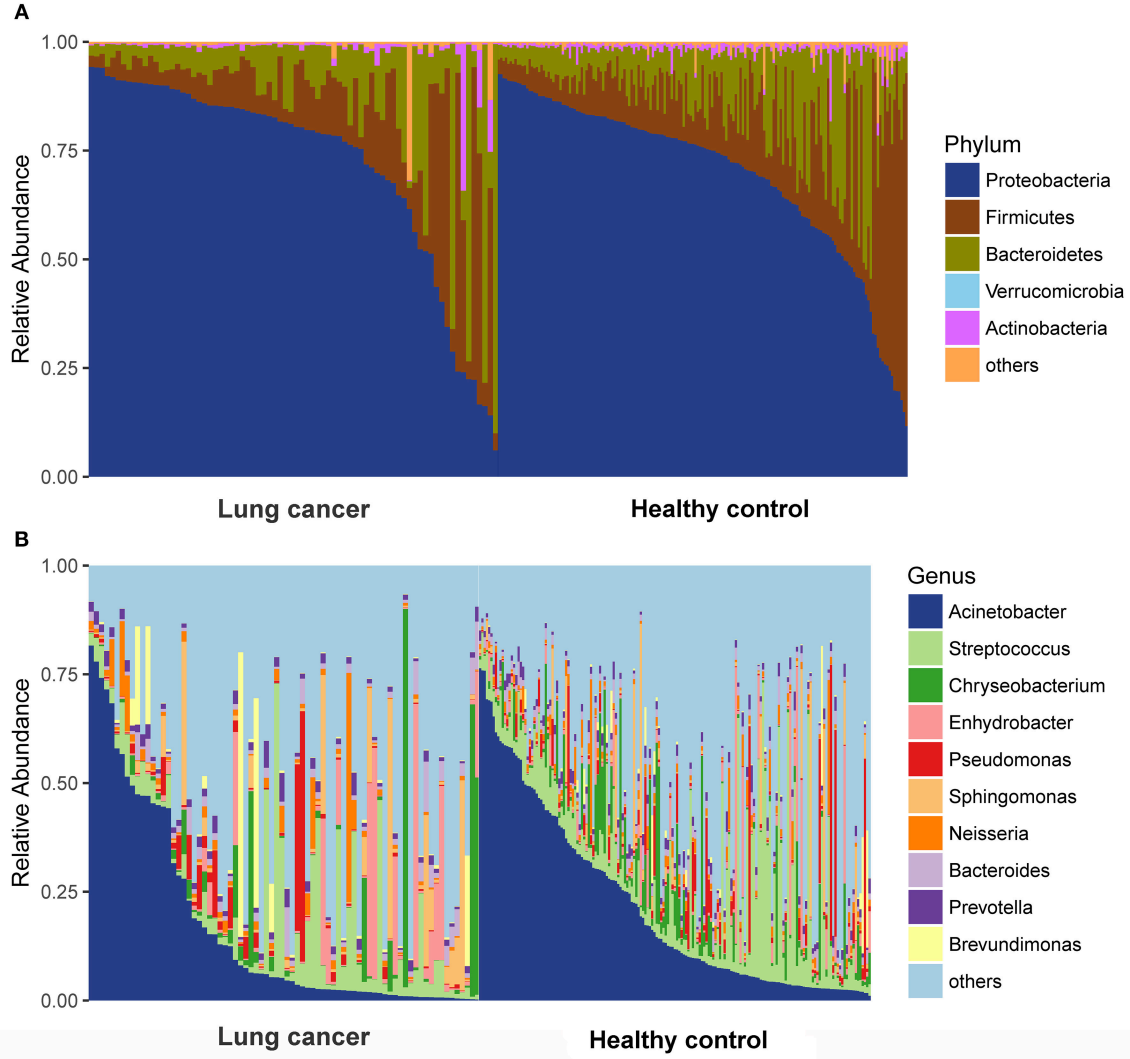
Taxonomic characterization of the salivary microbiome among lung cancer and control. **(A)** Phylum level; **(B)** Genus level.

**Figure 3:**
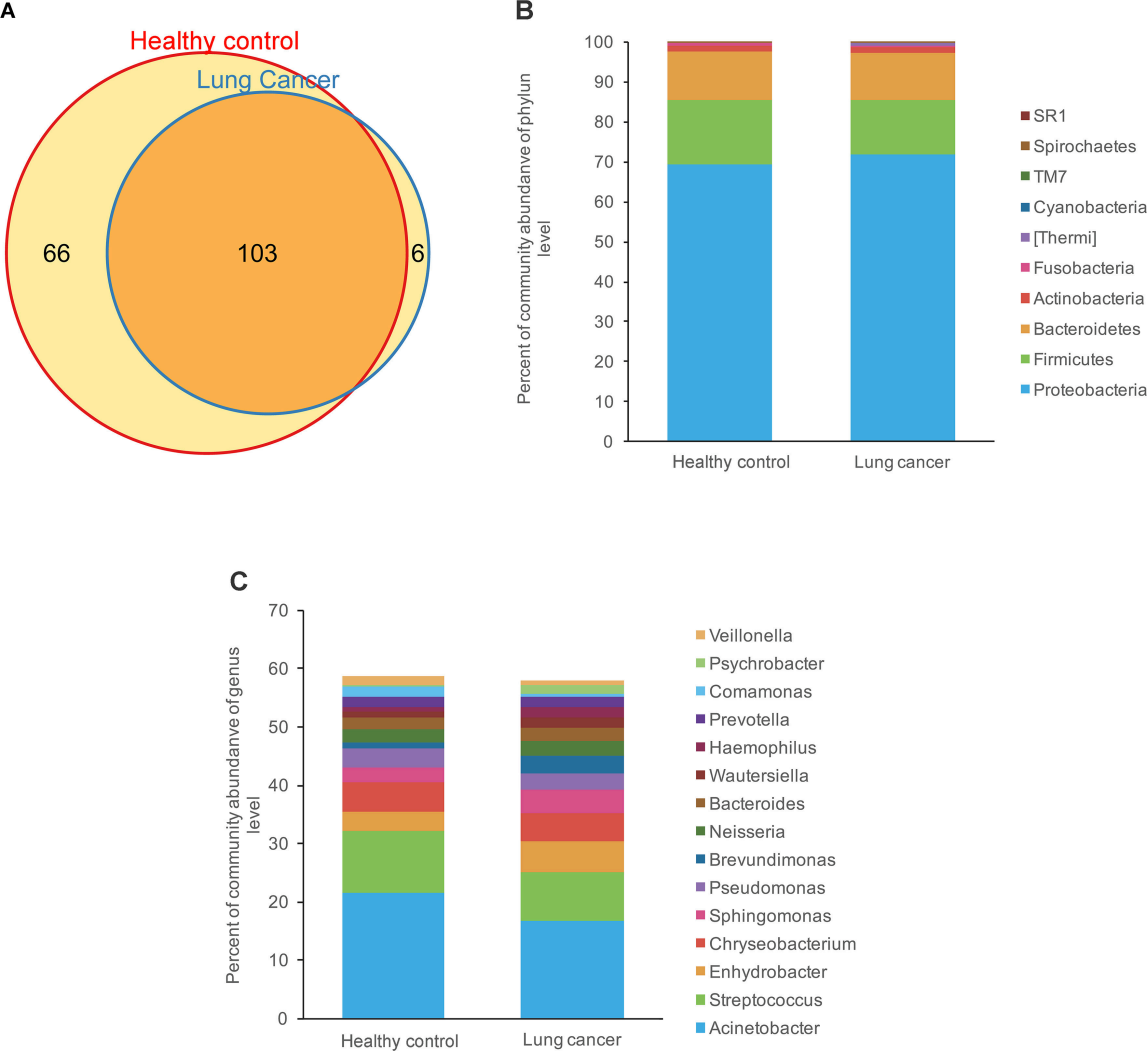
Comparison of OTUs and relative taxa abundance between lung cancer and control groups. **(A)** Venn diagram; **(B)** comparison of relative taxa abundance between lung cancer and control group at phylum level; **(C)** comparison of relative taxa abundance between lung cancer and control group at genus level.

We compared relative taxon abundance in the microbiota of lung cancer patients and control subjects, to further explore the oral microbial community features of lung cancer patients. Figure 2 illustrates the per subject bacterial taxonomy distribution at the genus and phylum level. It can be seen that the inter-individual variation in taxonomic composition is high and that no taxa is dominantly present among all individuals at the sequencing depth employed. Venn diagrams are used to evaluate the number and identity of the shared OTUs between the cancer and the control groups. The result indicates that 64.08% (66/103) and 5.82% (6/103) of the core OTUs were identified in these two groups (Figure 3A). At the phylum level, Proteobacteria, Firmicutes and Bacteroidetes were the dominant phyla representing over 97% of the total phyla in both the cancer and control groups. The microbiome composition was dominated by the phyla Proteobacteria (71.80% ± 23.22% in patients and 69.35% ± 18.97% (s.d.) in controls) and Firmicutes (13.74% ± 15.96% in patients and 16.22% ± 16.83% (s.d.) in controls), followed by Bacteriodetes (11.88% % ± 17.06% in patients and 11.93% ± 10.82% (s.d.) in controls; Figure 3B). The genus *Acinetobacter* (16.79% ± 22.70% in patients and 21.48% ± 20.42% (s.d.) in controls) dominated the microbiome, followed by *Streptococcus* with a high variation (8.19% ± 12.64% in patients and 10.76% ± 13.91% (s.d.) in controls). (Figure 3C).

### Significant variation of microbiome structure between lung cancer patients and controls

**Figure 4:**
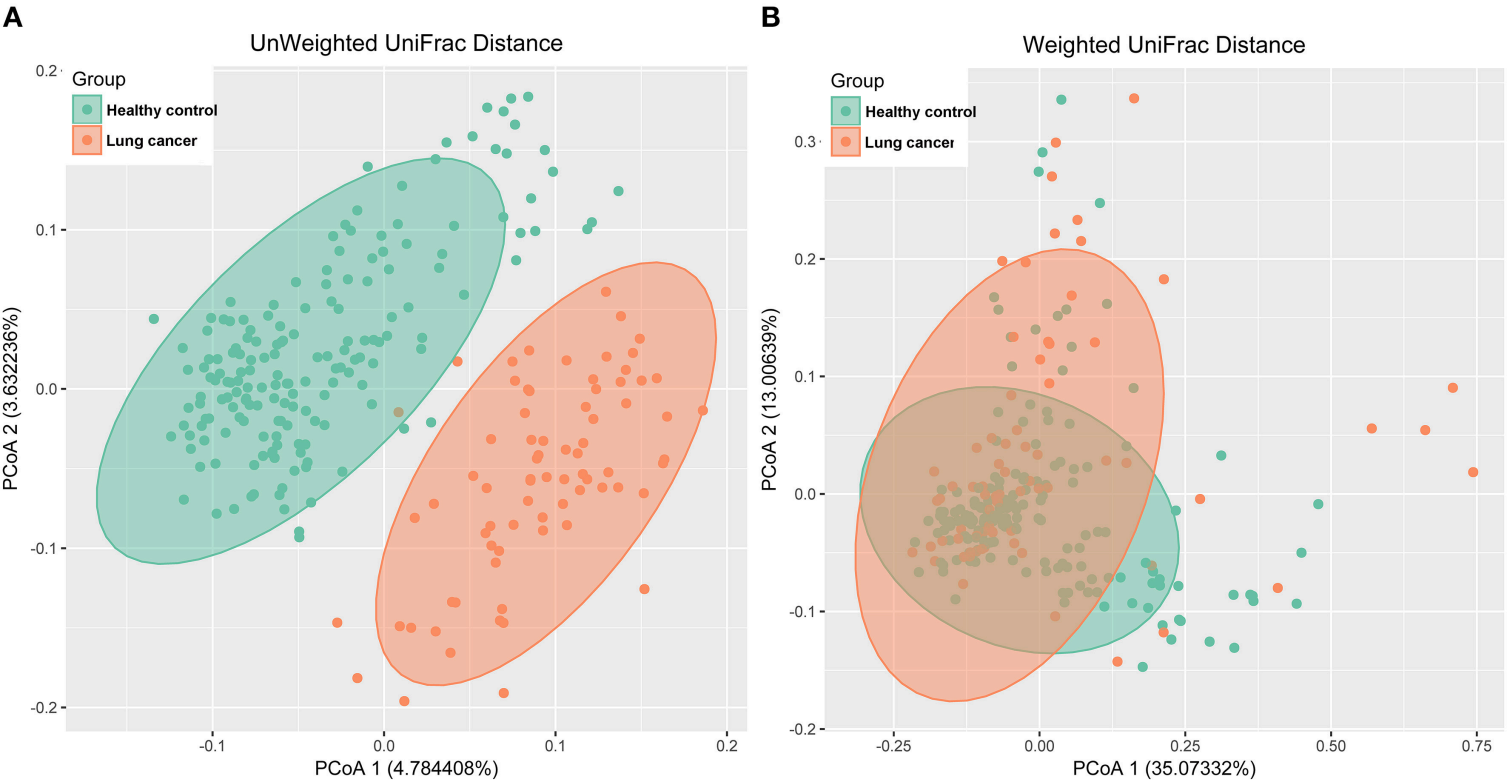
PCoA analysis of the microbiota among lung cancer and control groups. **(A)** unweighted UniFrac PCoA; **(B)** weighted UniFrac PCoA.

The principal coordinates analysis (PCoA) based on unweighted UniFrac distance (Figure 4A) and weighted UniFrac distance (Figure 4B) indicated a significant difference between the lung cancer and control groups, and the analysis of similarities (ANOSIM) indicated that the structure of the salivary microbiota significantly differed between the lung cancer and the control groups (ANOSIM, r = 0.454, *P* < 0.001, unweighted UniFrac; r = 0.113, *P* < 0.01, weighted UniFrac).

### Differential taxonomic abundance between lung cancer patients and controls

**Figure 5:**
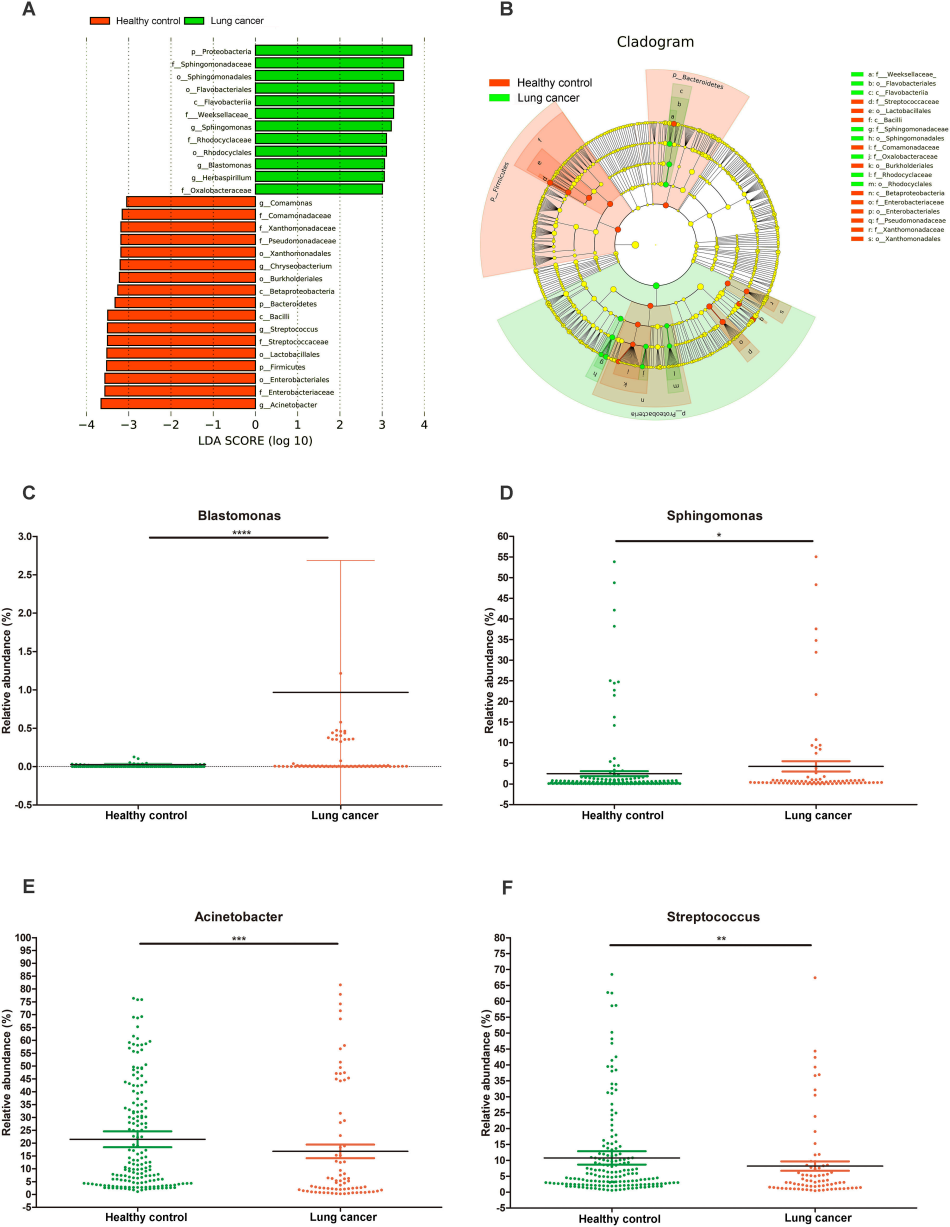
Characteristics of microbial community composition in lung cancer and control groups. **(A)** Enriched taxa in lung cancer and control oral microbiota are represented in a cladogram. The central point represents the root of the tree (bacteria), and each ring represents the next lower taxonomic level (phylum to genus: p, phylum; c, class; o, order; f, family; g, genus). The diameter of each circle represents the relative abundance of the taxon. **(B)** Most differentially abundant taxa between lung cancer and control groups (LDA score above 3), generated from LEfSe analysis. **(C–F)** Comparison of relative abundance at the bacterial genus level between lung cancer and control groups; *P < 0.05, **P < 0.01, *** *P* < 0.001, **** *P* < 0.0001 significantly different by Mann Whitney test.

A LEfSe comparison of the oral microbiota between the control and lung cancer groups was performed to study the specific lung cancer development-associated bacterial taxa. An LDA score above 3 indicated the greatest difference in taxa from the phylum to the genus level (Figure 5A). A cladogram is used to represent the structure and predominant bacteria of the microbiota in the control and lung cancer groups (Figure 5B). The level of Proteobacteria at the phylum level increased while the level of Firmicutes decreased, in the lung cancer group (Figure 5A). The genera *Sphingomonas* (*P* < 0.05) *and Blastomonas* (*P* < 0.0001; belonging to the family Sphingomonadaceae; Figures 5C,D) were enriched in non-smoking female lung cancer patients, whereas *Acinetobacter* (*P* < 0.001) and *Streptococcus* (*P* < 0.01) were enriched in the controls (Figures 5E,F).

### Associations between oral microbiota and immunocytochemistry markers

**Figure 6:**
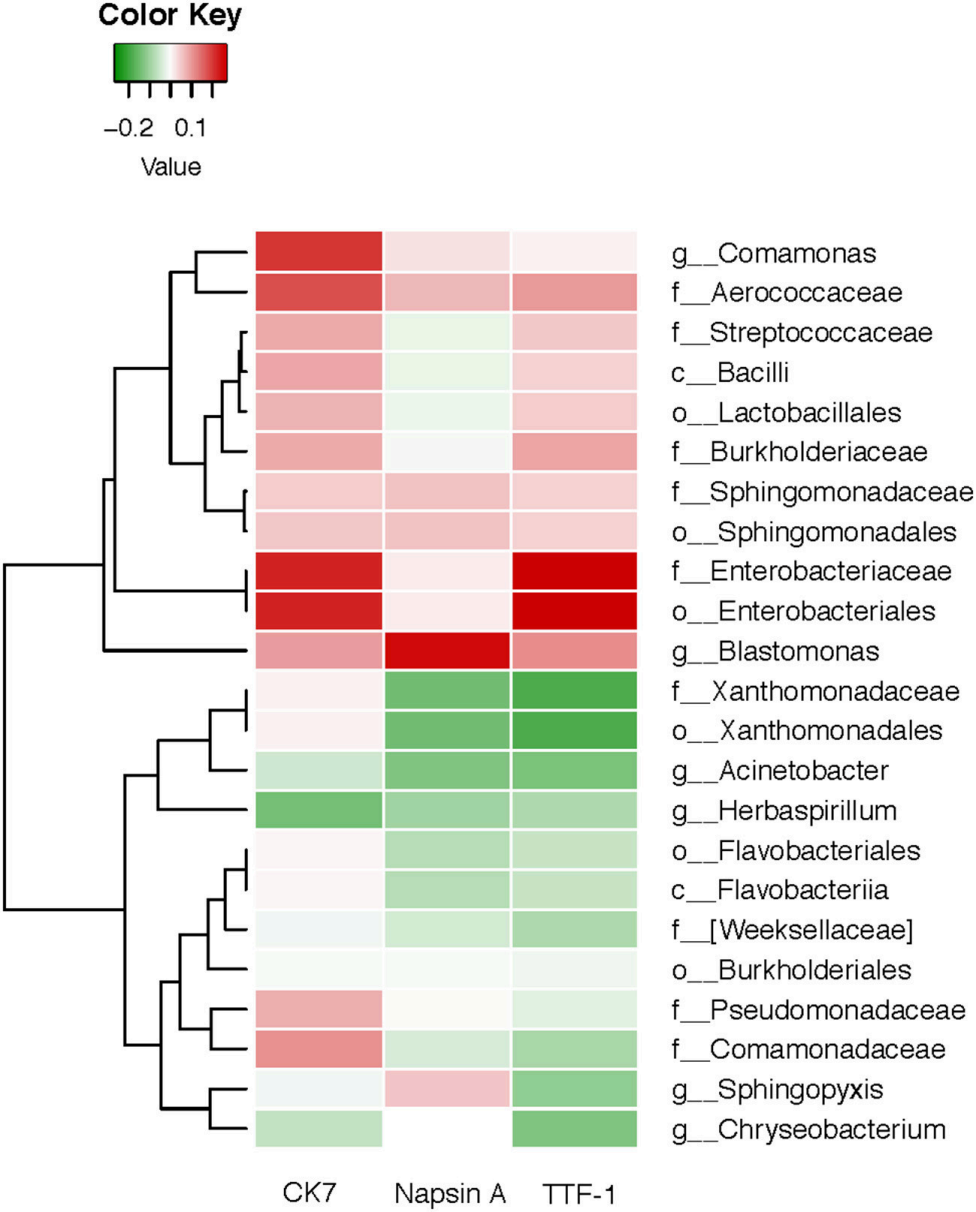
Heatmap for Spearman correlation analysis between oral microbiota of lung cancer and the immunocytochemistry markers.

We analyzed a set of immunocytochemistry markers for thyroid transcription factor (TTF-1), Napsin A and cytokeratin (CK7), and compared their results with the corresponding salivary microbiome. The Spearman's rank correlation coefficient was calculated for the oral microbiota of each subject and several immunohistochemical markers consisting of TIF-1, Napsin A, and CK7 (Figure 6). There were significant correlations (*P* < 0.05) among two bacterial biomarkers and the three immunohistochemical markers. As displayed in Figure 6, there is a significant positive correlation between CK7 and Enterobacteriaceae (r = 0.223, *P* < 0.05). At the same time, Napsin A is positively associated with genera *Blastomonas* (r = 0.251, *P* < 0.05). TTF-1 exhibits a significant positive (r = 0.262, *P* < 0.05) correlation with Enterobacteriaceae.

### Functional capacity changes of oral microbiome associated with lung cancer

**Figure 7:**
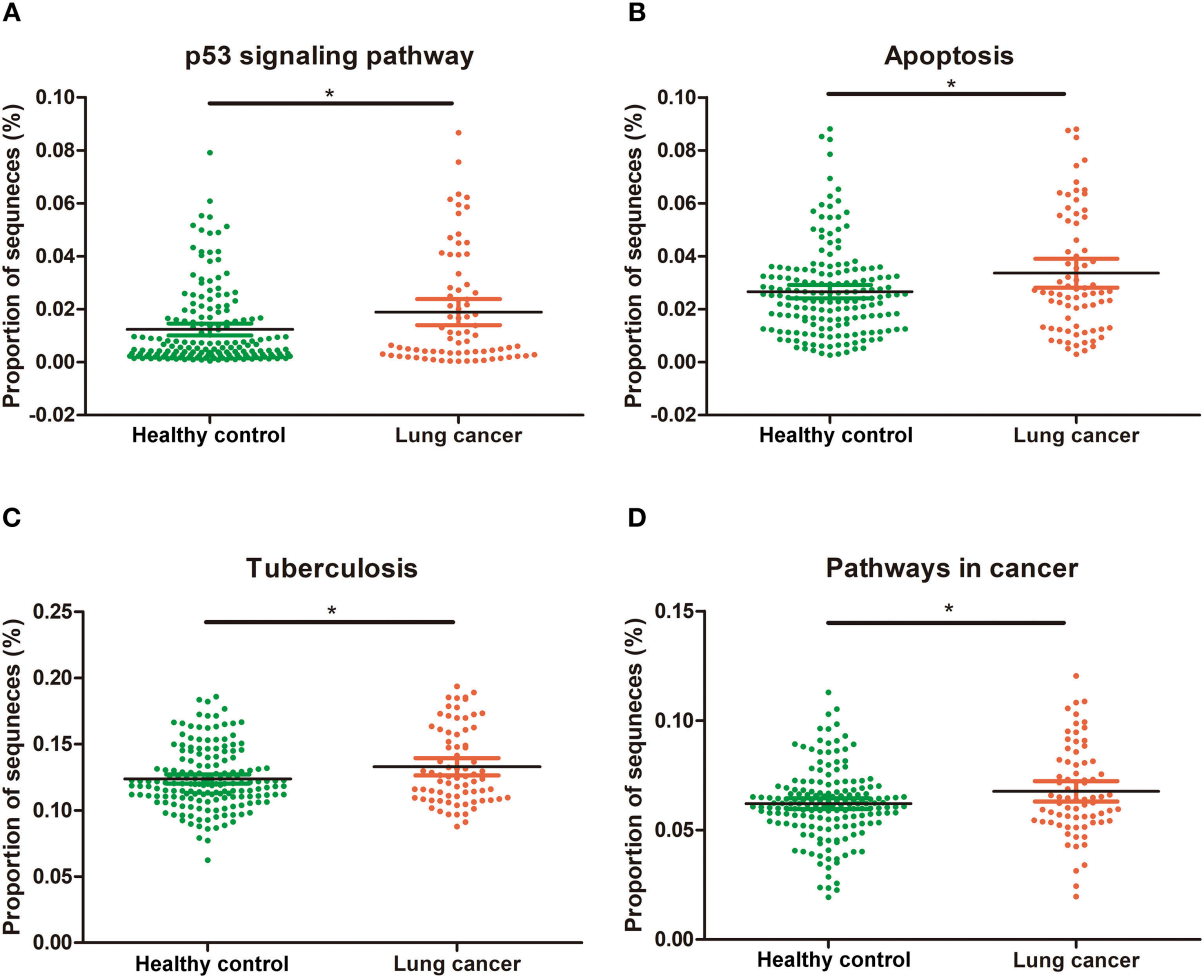
Functional categories with statistically significant differences between tumor and controls at level. **(A)** p53 signaling pathway; **(B)** apoptosis; **(C)** tuberculosis; **(D)** pathways in cancer. *P < 0.05.

Although 16S rRNA gene analysis indicate the presence of bacteria in a given sample, it does not provide information with respect to their functions. Hence, we used the PICRUSt program to analyze our data, to indirectly infer the function based on the known pathways of organisms categorized to a given species-level OTU. It was observed that functional pathways relating to cancer, p53 signaling pathway, apoptosis, and tuberculosis were enriched in non-smoking female lung cancer patients (Figure 7).

## Discussion

Our study revealed that salivary microbiota was significantly associated with lung cancer in non-smoking women. A high-throughput method was used to analyze bacterial populations in the saliva samples of non-smoking female lung cancer subjects and matched controls. It was found that there was lower microbial diversity and richness and obvious dysbiosis in the salivary microbiota of non-smoking female lung cancer patients, and the structure also differed when compared to that of the healthy controls. There is also evidence that certain clinical immunocytochemistry markers may be correlated with the variation in salivary microbiome. This is a pioneering investigation demonstrating that high-throughput measurements of salivary microbiome indicate potential bacterial dysbiosis associated with lung cancer in non-smoking women.

The salivary microbiota is dependent on the food consumption patterns, ethnic backgrounds, age (40) and oral environment, such as smoking (41). Several studies have observed effects of smoking on oral bacteria (21). Quite often, lung cancer is considered as a disease of smokers, however there have been limited efforts to study lung cancer in non-smokers (3, 42). Recently, major differences in terms of gender, and molecular and clinical-pathological discrepancy in non-smokers and smokers with lung cancer have been identified, indicating that they may not be the same disease (2, 12). It was also suggested that gender-dependent hormones could play a potential role in the development of lung cancer, based on the fact that lung cancer in female non-smokers exhibited a higher proportion compared with male non-smokers (5, 43). It is as yet unknown whether there is a greater contribution of risk factors other than smoking to the increased risk of carcinogenesis in non-smoking female patients (44). Non-smoking female lung cancer patients are considered a different population (45) and our unique study population is a major strength of our study, as the correlation between lung cancer and oral microbiome could be suitably studied because the effect of smoking was excluded.

The key findings of our study indicate that genera *Blastomonas* and *Sphingomonas* were significantly increased in the oral microbiota of lung cancer patients, while *Acinetobacte* and *Streptococcus* were higher in controls. Recent studies also identified *Streptococcus* in chronic obstructive pulmonary disease (COPD) (46) and cystic fibrosis (CF) lung microbiota (47). Under certain circumstance, *Streptococcus* could become potentially invasive to target the host fibronectin. Thereafter, a cytokine response could be induced to promote inflammation and further lead to carcinogenesis (48, 49). *Blastomonas* and *Sphingomonas* were previously identified as present in relatively high abundance in pneumonia patients (50). They also have a critical impact in systemic immune responses and affect the therapy for COPD and lung cancer (51, 52).

The dynamic balance between oral microbiota and the immune system has as yet not been fully studied. The healthy immune system in the oral cavity not only interacts with the commensal microbiota but also has to defend against pathogenic microbes (53). Oral microbiota can also be altered by immune dysregulation. There are several human diseases that could affect the balance of the host oral microbiota and effect the host immune system. When there is any disturbance in this balance of the immune system, the symbiotic relationship could shift, causing extensive colonization, and growth of conditioned pathogens. These opportunistic pathogens would induce pathogenic process that could finally lead to various symptomatic malignancies (53, 54).

In turn, the dysbiosis of oral microbiota affects the systemic immune system, which may intensify immune disorders. Therefore, oral bacteria could be involved in the pathogenesis and development of lung cancer (54). The microbial balance or “symbiosis” turns into imbalance or “dysbiosis” due to various influencing factors, which could probably contribute to the pathogenesis of the diseases through systemic inflammatory responses (55).

The Spearman's rank correlation analysis conducted in this study indicated that the oral microbiota was associated with immunocytochemistry markers in lung cancer. TTF-1 and CK7 exhibited a significantly positive correlation with Enterobacteriaceae, and Napsin A was positively associated with genus *Blastomonas*. The major advantage of this study is the novel hypothesis and the first report on the relationship between salivary microbiota and immunoreactivity markers in non-smoking female lung cancer patients. The results provide novel insights that are important for studies on salivary microbiome associated with lung cancer in non-smoking women, and which correlate with immunocytochemistry markers. TTF-1 is found in epithelial cells from thyroid and lung tumors. Studies have shown that TTF-1 could play a critical role in the pathogenesis of primary lung adenocarcinoma (25, 56). CK7 is clinically used to label certain types of normal and neoplastic glandular epithelia, which may be positive in lungs (57). As a member of the aspartic proteinase family, Napsin A is expressed in healthy lungs and is also frequently expressed in lung adenocarcinomas. Using immunohistochemistry for TTF-1, CK7 and Napsin A, we can reduce the false diagnosis rate due to aberrant immunoreactivity and thus increase reliability (58).

Our study found that TTF-1 and CK7 exhibited a significantly positive correlation with Enterobacteriaceae. As a significantly opportunistic pathogen, Enterobacteriaceae can exist in the human gut without causing symptoms or diseases under normal conditions (59). Therefore, significant variation of Enterobacteriaceae may be caused by host immunity and environmental factors such as redox state and oxygen availability, which may lead to immune responses to disease development in the case of microbiota dysbiosis in the lung (60). A series of recent studies have started to focus on gut microbiota in lung disease and gut-lung axis (61–63). These new findings accumulated to better understand the links between microbial exposure and autoimmunity and allergy (60).

Our study also found that Napsin A was positively associated with genus *Blastomonas*. The genus *Blastomonas*, which belongs to the family Sphingomonadaceae, could be isolated from lake water, sea water, fresh water, and even hospital water (64–66). Even though disinfectants are used in drinking water distribution systems (DWDS), plumbing systems and fixtures in buildings are colonized by bacteria (67). Drinking water could influence the composition and diversity of commensal oral and gut bacteria in human, inducing an altered autoimmune response and lung diseases incidence (68).

There are complex interactions between oral microbiota and the host, and as yet, our knowledge about these comprehensive interactions is limited (60). However, it is not necessarily that the functions of the microbiota are solely dependent on any one of these interactions, and alterations in these relationships may affect human health and cause disease (60). These bacteria may shed different microbial bioactive molecules and affect the host (69). It is known that apoptosis is highly regulated and plays an important role in immune response and tumorigenesis (70, 71). The p53 signaling pathway is recognized as a potential risk factor in lung adenocarcinoma tumorigenesis and survival (72). The observed up-regulation in the p53 signaling pathways suggests potential pathogenic functions of these salivary microbiota. These findings suggest that oral microbiome potentially regulate of lung cancer cell apoptosis through p53 pathway, albeit it is not the exclusive pathway in this process.

Further longitudinal studies need to be conducted to testify whether microbiome variation is a causative factor for carcinogenesis or an abductive consequence of cancer onset (73). Salivary microbiota may influence secondary metabolism and participate in immunity and stress resistance of the host (74). Whether or not it is viable, oral microbiota could still play an important role in inflammatory responses (60). In contrast, oral microorganisms are more likely to be colonize and overgrowth in the respiratory tract of subjects with lung disease (75). Different types of bacteria may contribute to the host-microbe interactions in various manner, which urgently demand future investigations to decipher the mechanisms of each of these associations (60).

In summary, this study provides an insight on oral microbiome as a potential reservoir of bacterial pathogens in non-smoking female lung cancer (76). Discovery of potential relationship between host and bacterial biomarkers may lead to non-invasive strategies to help detecting and classifying the different disease stages. Goals include identifying new host and microbial biomarkers and function, characterizing the function of the host innate and adaptive microbial metabolism systems, and the dynamic operations of the microbiota.

## Conclusions

This study suggests the critical role microbiota dysbiosis plays and indicates that certain bacterial species may contribute to lung cancer in non-smoking women. These results reveal distinct salivary microbiome profiles in non-smoking female lung cancer patients and provide evidence that salivary microbiota can be an informative source for discovering non-invasive biomarkers of lung cancer.

## Ethics statement

This study was conducted as per the recommendations of the Human Specimen Study guidelines of the Institutional Review Board of Affiliated Central Hospital of Qingdao University (IRB# QCH16-1101-01), with written informed consent taken from all subjects. All subjects gave written informed consent in accordance with the Declaration of Helsinki. The protocol was approved by the Institutional Review Board of Affiliated Central Hospital of Qingdao University.

## Data availability

All sequencing data associated with this study were uploaded to the NCBI SRA database (accession number: SUB 4019113). The SRA database webpage is https://www.ncbi.nlm.nih.gov/sra/.

## Author contributions

LZ and CZhang designed the study. JY, JZ, BC, CZhao, JW, and CL performed measurements and data analysis. XM, YW, DZ, ZZ, and XH obtained samples and clinical details. JY and LZ wrote the manuscript. All authors have read and critically revised the manuscript.

### Conflict of interest statement

The authors declare that the research was conducted in the absence of any commercial or financial relationships that could be construed as a potential conflict of interest.

## References

[1] I H Cho JY. Lung cancer biomarkers. Adv Clin Chem. (2015) 72:107–70. 10.1016/bs.acc.2015.07.003 26471082

[2] Sun S Schiller JH Gazdar AF. Lung cancer in never smokers–a different disease. Nat Rev Cancer (2007) 7:778–90. 10.1038/nrc2190 17882278

[3] Kim JH Park K Yim SH Choi JE Sung JS Park JY. Genome-wide association study of lung cancer in Korean non-smoking women. J Korean Med Sci. (2013) 28:840–7. 10.3346/jkms.2013.28.6.840 23772147 PMC3677999

[4] Govindan R Ding L Griffith M Subramanian J Dees ND Kanchi KL. Genomic landscape of non-small cell lung cancer in smokers and never-smokers. Cell (2012) 150:1121–34. 10.1016/j.cell.2012.08.024 22980976 PMC3656590

[5] Lan Q Hsiung CA Matsuo K Hong YC Seow A Wang Z. Genome-wide association analysis identifies new lung cancer susceptibility loci in never-smoking women in Asia. Nat Genet. (2012) 44:1330–5. 10.1038/ng.2456 23143601 PMC4169232

[6] Chen H Jiang W. Application of high-throughput sequencing in understanding human oral microbiome related with health and disease. Front Microbiol. (2014) 5:508. 10.3389/fmicb.2014.00508 25352835 PMC4195358

[7] Shoemark DK Allen SJ. The microbiome and disease: reviewing the links between the oral microbiome, aging, and Alzheimer's disease. J Alzheimers Dis. (2015) 43:725–38. 10.3233/JAD-141170 25125469

[8] Zhang X Zhang D Jia H Feng Q Wang D Liang D. The oral and gut microbiomes are perturbed in rheumatoid arthritis and partly normalized after treatment. Nat Med. (2015) 21:895–905. 10.1038/nm.3914 26214836

[9] Li J Hao C Ren L Xiao Y Wang J Qin X. Data mining of lung microbiota in cystic fibrosis patients. PLoS ONE (2016) 11:e0164510. 10.1371/journal.pone.0164510 27741283 PMC5065158

[10] Long J Cai Q Steinwandel M Hargreaves MK Bordenstein SR Blot WJ. Association of oral microbiome with type 2 diabetes risk. J Periodontal Res. (2017) 52:636–43. 10.1111/jre.12432 28177125 PMC5403709

[11] Shukla SD Budden KF Neal R Hansbro PM. Microbiome effects on immunity, health and disease in the lung. Clin Transl Immunol. (2017) 6:e133. 10.1038/cti.2017.6 28435675 PMC5382435

[12] Sun J Mechanisms Underlying Host-Microbiome Interactions in Pathophysiology of Human Diseases. New York, NY: Springer Science+Business Media (2018). 10.1007/978-1-4939-7534-1

[13] Marsland BJ Gollwitzer ES. Host-microorganism interactions in lung diseases. Nat Rev Immunol. (2014) 14:827–35. 10.1038/nri3769 25421702

[14] Krishnan K Chen T Paster BJ. A practical guide to the oral microbiome and its relation to health and disease. Oral Dis. (2017) 23:276–86. 10.1111/odi.12509 27219464 PMC5122475

[15] Segal LN Alekseyenko AV Clemente JC Kulkarni R Wu B Gao Z. Enrichment of lung microbiome with supraglottic taxa is associated with increased pulmonary inflammation. Microbiome (2013) 1:19. 10.1186/2049-2618-1-19 24450871 PMC3971609

[16] Segal LN Clemente JC Tsay JC Koralov SB Keller BC Wu BG. Enrichment of the lung microbiome with oral taxa is associated with lung inflammation of a Th17 phenotype. Nat Microbiol. (2016) 1:16031. 10.1038/nmicrobiol.2016.31 27572644 PMC5010013

[17] Dewhirst FE Chen T Izard J Paster BJ Tanner AC Yu WH. The human oral microbiome. J Bacteriol. (2010) 192:5002–17. 10.1128/JB.00542-10 20656903 PMC2944498

[18] Yan X Yang M Liu J Gao R Hu J Li J. Discovery and validation of potential bacterial biomarkers for lung cancer. Am J Cancer Res. (2015) 5:3111–22. 26693063 PMC4656734

[19] Gomez A Nelson KE. The oral microbiome of children: development, disease, and implications beyond oral health. Microb Ecol. (2017) 73:492–503. 10.1007/s00248-016-0854-1 27628595 PMC5274568

[20] Kumar PS. From focal sepsis to periodontal medicine: a century of exploring the role of the oral microbiome in systemic disease. J Physiol. (2017) 595:465–76. 10.1113/JP272427 27426277 PMC5233655

[21] Wu J Peters BA Dominianni C Zhang Y Pei Z Yang L. Cigarette smoking and the oral microbiome in a large study of American adults. ISME J. (2016) 10:2435–46. 10.1038/ismej.2016.37 27015003 PMC5030690

[22] Huber RM. Is lung cancer in never-smokers a different disease?–Back to the figures. J Thorac Oncol. (2007) 2:787–8. 10.1097/JTO.0b013e318153f3c,5 17805053

[23] Choi JR Park SY Noh OK Koh YW Kang DR. Gene mutation discovery research of non-smoking lung cancer patients due to indoor radon exposure. Ann Occup Environ Med. (2016) 28:13. 10.1186/s40557-016-0095-2 26985396 PMC4793700

[24] Ao MH Zhang H Sakowski L Sharma R Illei PB Gabrielson E. The utility of a novel triple marker (combination of TTF1, napsin A, and p40) in the subclassification of non-small cell lung cancer. Hum Pathol. (2014) 45:926–34. 10.1016/j.humpath.2014.01.005 24746197 PMC4178947

[25] Kawai T Tominaga S Hiroi S Kameda K Ogata S Nakashima H. Expressions of thyroid transcription factor-1, Napsin A, p40, p63, CK5/6 and Desmocollin-3 in non-small cell lung cancer, as revealed by imprint cytology using a malinol-based cell-transfer technique. Acta Cytol. (2015) 59:457–64. 10.1159/000442659 26696549

[26] Pepe MS Feng Z Janes H Bossuyt PM Potter JD. Pivotal evaluation of the accuracy of a biomarker used for classification or prediction: standards for study design. J Natl Cancer Inst. (2008) 100:1432–8. 10.1093/jnci/djn326 18840817 PMC2567415

[27] Zhang L Farrell JJ Zhou H Elashoff D Akin D Park NH. Salivary transcriptomic biomarkers for detection of resectable pancreatic cancer. Gastroenterology (2010) 138:949–57 e941–947. 10.1053/j.gastro.2009.11.010 19931263 PMC2831159

[28] Farrell JJ Zhang L Zhou H Chia D Elashoff D Akin D. Variations of oral microbiota are associated with pancreatic diseases including pancreatic cancer. Gut (2012) 61:582–8. 10.1136/gutjnl-2011-300784 21994333 PMC3705763

[29] Zhao W Wang H Peng Y Tian B Peng L Zhang DC. DeltaNp63, CK5/6, TTF-1 and napsin A, a reliable panel to subtype non-small cell lung cancer in biopsy specimens. Int J Clin Exp Pathol. (2014) 7:4247–53. 25120805 PMC4129040

[30] Chen R Ding Z Zhu L Lu S Yu Y. Correlation of clinicopathologic features and lung squamous cell carcinoma subtypes according to the 2015 WHO classification. Eur J Surg Oncol. (2017) 43:2308–14. 10.1016/j.ejso.2017.09.011 28964610

[31] Woese CR Fox GE. Phylogenetic structure of the prokaryotic domain: the primary kingdoms. Proc Natl Acad Sci USA (1977) 74:5088–90. 10.1073/pnas.74.11.5088 270744 PMC432104

[32] Woese CR Kandler O Wheelis ML. Towards a natural system of organisms: proposal for the domains Archaea, Bacteria, and Eucarya. Proc Natl Acad Sci USA (1990) 87:4576–9. 10.1073/pnas.87.12.4576 2112744 PMC54159

[33] Magoc T Salzberg SL. FLASH: fast length adjustment of short reads to improve genome assemblies. Bioinformatics (2011) 27:2957–63. 10.1093/bioinformatics/btr507 21903629 PMC3198573

[34] Caporaso JG Kuczynski J Stombaugh J Bittinger K Bushman FD Costello EK. QIIME allows analysis of high-throughput community sequencing data. Nat Methods (2010) 7:335–6. 10.1038/nmeth.f.303 20383131 PMC3156573

[35] Edgar RC. Search and clustering orders of magnitude faster than BLAST. Bioinformatics (2010) 26:2460–1. 10.1093/bioinformatics/btq461 20709691

[36] Cole JR Wang Q Cardenas E Fish J Chai B Farris RJ. The Ribosomal Database Project: improved alignments and new tools for rRNA analysis. Nucleic Acids Res. (2009) 37:D141–145. 10.1093/nar/gkn879 19004872 PMC2686447

[37] Segata N Izard J Waldron L Gevers D Miropolsky L Garrett WS. Metagenomic biomarker discovery and explanation. Genome Biol. (2011) 12:R60. 10.1186/gb-2011-12-6-r60 21702898 PMC3218848

[38] Langille MG Zaneveld J Caporaso JG Mcdonald D Knights D Reyes JA. Predictive functional profiling of microbial communities using 16S rRNA marker gene sequences. Nat Biotechnol. (2013) 31:814–21. 10.1038/nbt.2676 23975157 PMC3819121

[39] Parks DH Beiko RG. Identifying biologically relevant differences between metagenomic communities. Bioinformatics (2010) 26:715–21. 10.1093/bioinformatics/btq041 20130030

[40] Mason MR Nagaraja HN Camerlengo T Joshi V Kumar PS. Deep sequencing identifies ethnicity-specific bacterial signatures in the oral microbiome. PLoS ONE (2013) 8:e77287. 10.1371/journal.pone.0077287 24194878 PMC3806732

[41] Chaouachi K Sajid KM. A critique of recent hypotheses on oral (and lung) cancer induced by water pipe (hookah, shisha, narghile) tobacco smoking. Med Hypotheses (2010) 74:843–6. 10.1016/j.mehy.2009.11.036 20036075

[42] Listed N Lung cancer: not just for smokers. People who never smoked may have a different form of the disease that responds better to a new generation of targeted medications. Harvard Health Letter (2007) 32:4. 17323498

[43] Saito S Espinoza-Mercado F Liu H Sata N Cui X Soukiasian HJ. Current status of research and treatment for non-small cell lung cancer in never-smoking females. Cancer Biol Ther. (2017) 18:359–68. 10.1080/15384047.2017.1323580 28494184 PMC5536938

[44] Gazdar AF Thun MJ. Lung cancer, smoke exposure, and sex. J Clin Oncol. (2007) 25:469–71. 10.1200/JCO.2006.09.4623 17290053

[45] Yano T Haro A Shikada Y Maruyama R Maehara Y. Non-small cell lung cancer in never smokers as a representative 'non-smoking-associated lung cancer': epidemiology and clinical features. Int J Clin Oncol. (2011) 16:287–93. 10.1007/s10147-010-0160-8 21562939

[46] Pragman AA Lyu T Baller JA Gould TJ Kelly RF Reilly CS. The lung tissue microbiota of mild and moderate chronic obstructive pulmonary disease. Microbiome (2018) 6:7. 10.1186/s40168-017-0381-4 29316977 PMC5759273

[47] Coburn B Wang PW Diaz Caballero J Clark ST Brahma V Donaldson S. Lung microbiota across age and disease stage in cystic fibrosis. Sci Rep. (2015) 5:10241. 10.1038/srep10241 25974282 PMC4431465

[48] Erb-Downward JR Thompson DL Han MK Freeman CM Mccloskey L Schmidt LA. Analysis of the lung microbiome in the “healthy” smoker and in COPD. PLoS ONE (2011) 6:e16384. 10.1371/journal.pone.0016384 21364979 PMC3043049

[49] Liu HX Tao LL Zhang J Zhu YG Zheng Y Liu D. Difference of lower airway microbiome in bilateral protected specimen brush between lung cancer patients with unilateral lobar masses and control subjects. Int J Cancer (2018) 142:769–78. 10.1002/ijc.31098 29023689

[50] Heirali A Mckeon S Purighalla S Storey DG Rossi L Costilhes G. Assessment of the microbial constituents of the home environment of individuals with cystic fibrosis (CF) and their association with lower airways infections. PLoS ONE (2016) 11:e0148534. 10.1371/journal.pone.0148534 26859493 PMC4747485

[51] Zhang Y Sun J Lin CC Abemayor E Wang MB Wong DT. The emerging landscape of salivary diagnostics. Periodontol (2016) 70:38–52. 10.1111/prd.12099 26662481

[52] Mayhew D Devos N Lambert C Brown JR Clarke SC Kim VL. Longitudinal profiling of the lung microbiome in the AERIS study demonstrates repeatability of bacterial and eosinophilic COPD exacerbations. Thorax (2018) 73:422–30. 10.1136/thoraxjnl-2017-210408 29386298 PMC5909767

[53] Idris A Hasnain SZ Huat LZ Koh D Human diseases, immunity and the oral microbiota—Insights gained from metagenomic studies. Oral Science Int. (2017) 14:27–32. 10.1016/S1348-8643(16)30024-6

[54] Xun Z Zhang Q Xu T Chen N Chen F. Dysbiosis and ecotypes of the salivary microbiome associated with inflammatory bowel diseases and the assistance in diagnosis of diseases using oral bacterial profiles. Front Microbiol. (2018) 9:1136. 10.3389/fmicb.2018.01136 29899737 PMC5988890

[55] Brenner DR Mclaughlin JR Hung RJ. Previous lung diseases and lung cancer risk: a systematic review and meta-analysis. PLoS ONE (2011) 6:e17479. 10.1371/journal.pone.0017479 21483846 PMC3069026

[56] Iqbal J. Role of Napsin A, and TTF1 as a diagnostic marker for lung adenocarcinoma. Arch Pathol Lab Med. (2013) 137:155. 10.5858/arpa.2012-0123-LE 23368855

[57] Su YC Hsu YC Chai CY. Role of TTF-1, CK20, and CK7 immunohistochemistry for diagnosis of primary and secondary lung adenocarcinoma. Kaohsiung J Med Sci. (2006) 22:14–9. 10.1016/S1607-551X(09)70214-1 16570563 PMC11918202

[58] Jafarian AH Gharib M Mohammadian Roshan N Sherafatnia S Omidi AA Bagheri S. The diagnostic value of TTF-1, P63, HMWK, CK7, and CD56 immunostaining in the classification of lung Carcinoma. Iran J Pathol. (2017) 12:195–201. 29531543 PMC5835366

[59] Taur Y Pamer EG. Microbiome mediation of infections in the cancer setting. Genome Med. (2016) 8:40. 10.1186/s13073-016-0306-z 27090860 PMC4835935

[60] Budden KF Gellatly SL Wood DL Cooper MA Morrison M Hugenholtz P. Emerging pathogenic links between microbiota and the gut-lung axis. Nat Rev Microbiol. (2017) 15:55–63. 10.1038/nrmicro.2016.142 27694885

[61] Marsland BJ Trompette A Gollwitzer ES. The gut-lung axis in respiratory disease. Ann Am Thorac Soc. (2015) 12 (Suppl. 2):S150–6. 10.1513/AnnalsATS.201503-133AW 26595731

[62] Bingula R Filaire M Radosevic-Robin N Bey M Berthon JY Bernalier-Donadille A. Desired turbulence? gut-lung axis, immunity, and lung cancer. J Oncol. (2017) 2017:5035371. 10.1155/2017/5035371 29075294 PMC5623803

[63] Bradley CP Teng F Felix KM Sano T Naskar D Block KE. Segmented filamentous bacteria provoke lung autoimmunity by inducing gut-lung axis Th17 cells expressing dual TCRs. Cell Host Microbe (2017) 22:697–704 e694. 10.1016/j.chom.2017.10.007 29120746 PMC5749641

[64] Zeng Y Koblizek M Feng F Liu Y Wu Z Jian J. Whole-genome sequences of an aerobic anoxygenic phototroph, blastomonas sp. strain AAP53, isolated from a freshwater desert lake in inner mongolia, China. Genome Announc. (2013) 1:e0007113. 10.1128/genomeA.00071-13 23516202 PMC3622956

[65] Xiao N Liu Y Liu X Gu Z Jiao N Liu H. *Blastomonas aquatica* sp. nov, a bacteriochlorophyll-containing bacterium isolated from lake water. Int J Syst Evol Microbiol. (2015) 65:1653–8. 10.1099/ijs.0.000153 25724744

[66] Meng YC Liu HC Kang YQ Zhou YG Cai M. *Blastomonas marina* sp. nov, a bacteriochlorophyll-containing bacterium isolated from seawater. Int J Syst Evol Microbiol. (2017) 67:3015–9. 10.1099/ijsem.0.002070 28820113

[67] Soto-Giron MJ Rodriguez RL Luo C Elk M Ryu H Hoelle J. Biofilms on hospital shower hoses: characterization and implications for nosocomial infections. Appl Environ Microbiol. (2016) 82:2872–83. 10.1128/AEM.03529-15 26969701 PMC4836434

[68] Sofi MH Gudi R Karumuthil-Melethil S Perez N Johnson BM Vasu C. pH of drinking water influences the composition of gut microbiome and type 1 diabetes incidence. Diabetes (2014) 63:632–44. 10.2337/db13-0981 24194504 PMC3900548

[69] Tsay JJ Wu BG Badri MH Clemente JC Shen N Meyn P Airway microbiota is associated with up-regulation of the PI3K pathway in lung cancer. Am J Respir Crit Care Med. (2018). 10.1164/rccm.201710-2118OC PMC6221574 29864375

[70] Li RZ Fan XX Duan FG Jiang ZB Pan HD Luo LX. Proscillaridin A induces apoptosis and suppresses non-small-cell lung cancer tumor growth via calcium-induced DR4 upregulation. Cell Death Dis. (2018) 9:696. 10.1038/s41419-018-0733-4 29899551 PMC5999972

[71] Losuwannarak N Sritularak B Chanvorachote P. Cycloartobiloxanthone induces human lung cancer cell apoptosis via mitochondria-dependent apoptotic pathway. In Vivo (2018) 32:71–8. 10.21873/invivo.11206 29275301 PMC5892644

[72] Xu S Liu R Da Y. Comparison of tumor related signaling pathways with known compounds to determine potential agents for lung adenocarcinoma. Thorac Cancer (2018) 9:974–88. 10.1111/1759-7714.12773 29870138 PMC6068465

[73] Liu J Duan Y. Saliva: a potential media for disease diagnostics and monitoring. Oral Oncol. (2012) 48:569–77. 10.1016/j.oraloncology.2012.01.021 22349278

[74] Xiao H Zhang L Zhou H Lee JM Garon EB Wong DT. Proteomic analysis of human saliva from lung cancer patients using two-dimensional difference gel electrophoresis and mass spectrometry. Mol Cell Proteomics (2012) 11:M111 012112. 10.1074/mcp.M111.012112 22096114 PMC3277759

[75] Gao K Zhou H Zhang L Lee JW Zhou Q Hu S. Systemic disease-induced salivary biomarker profiles in mouse models of melanoma and non-small cell lung cancer. PLoS ONE (2009) 4:e5875. 10.1371/journal.pone.0005875 19517020 PMC2691577

[76] Thun MJ Hannan LM Adams-Campbell LL Boffetta P Buring JE Feskanich D. Lung cancer occurrence in never-smokers: an analysis of 13 cohorts and 22 cancer registry studies. PLoS Med. (2008) 5:e185. 10.1371/journal.pmed.0050185 18788891 PMC2531137

