# Supplementary material for: Dysbiosis of the Salivary Microbiome Is Associated With Non-smoking Female Lung Cancer and Correlated With Immunocytochemistry Markers
Source: Front Oncol. 2018 Nov 20;8:520. doi: 10.3389/fonc.2018.00520 (PMC6256243; doi:10.3389/fonc.2018.00520)
Supplement: Supplementary file 1 [file Data_Sheet_1.PDF]

Table.1 Clinical characteristics of samples and subjects of immunocytochemistry markers

Num.	Healthy control	Lung cancer			
	Age	Age	immunocytochemistry markers		
			CK7	Napsin A	TTF-1
1	59	51	3+	3+	2+
2	43	69	2+	2+	2+
3	58	41	+	+	+
4	52	64	+	+	+
5	46	74	+	+	+
6	66	75	+	+	+
7	57	59	+	+	+
8	58	77	+	+	+
9	47	61	+	+	+
10	57	70	2+	+	+
11	51	71	+	+	+
12	63	74	+	+	+
13	69	61	+	-	+
14	64	52	2+	+	+
15	57	70	+	+	+
16	60	63	+	+	+
17	59	62	+	+	+
18	57	80	+	+	+
19	51	53	+	+	+
20	76	53	+	+	+
21	64	72	+	+	+
22	55	66	+	+	+
23	66	53	+	+	+
24	59	64	+	+	+
25	47	76	+	+	+
26	56	59	+	+	+
27	60	60	3+	3+	3+
28	80	55	+	+	+
29	58	51	+	+	+
30	46	52	+	+	+
31	57	58	+	+	+
32	59	64	+	+	+
33	48	55	+	+	+
34	64	50	+	+	+
35	55	66	+	+	+
36	52	55	+	+	+
37	54	58	3+	-	3+
38	50	38	+	+	+

Table 1 cont'd

Num.	Healthy control	Lung cancer			
	Age	Age	immunocytochemistry markers		
			CK7	Napsin A	TTF-1
39	56	51	+	+	+
40	69	69	+	+	+
41	67	41	+	+	+
42	68	64	+	+	+
43	59	74	+	+	+
44	62	75	+	+	+
45	49	59	+	+	+
46	49	77	+	+	+
47	45	61	+	+	+
48	77	70	+	+	+
49	67	71	3+	2+	3+
50	54	74	+	+	+
51	63	61	+	+	+
52	50	52	+	+	+
53	54	70	2+	+	+
54	65	63	+	+	+
55	66	62	+	+	+
56	81	70	+	-	+
57	77	53	2+	+	+
58	64	53	+	+	+
59	55	72	+	+	+
60	66	66	+	+	+
61	59	53	+	+	+
62	47	64	+	+	+
63	56	76	+	+	+
64	60	59	+	+	+
65	80	60	+	+	+
66	45	59	+	+	+
67	51	60	2+	3+	2+
68	63	55	+	2+	+
69	68	51	+	+	+
70	64	52	+	+	+
71	54	58	+	+	+
72	63	64	+	+	+
73	48	55	+	+	+
74	57	50	+	+	+
75	52	55	+	+	+

Table 1 cont'd

Num.	Healthy control	Lung cancer			
	Age	Age	immunocytochemistry markers		
			CK7	Napsin A	TTF-1
76	66				
77	59				
78	57				
79	56				
80	60				
81	80				
82	58				
83	56				
84	57				
85	59				
86	58				
87	65				
88	55				
89	52				
90	55				
91	50				
92	56				
93	69				
94	67				
95	68				
96	59				
97	62				
98	59				
99	59				
100	55				
101	77				
102	65				
103	55				
104	66				
105	59				
106	57				
107	56				
108	60				
109	80				
110	55				
111	51				
112	63				

Table 1 cont'd

Num.	Healthy control	Lung cancer			
	Age	Age	immunocytochemistry markers		
			CK7	Napsin A	TTF-1
113	68				
114	65				
115	55				
116	63				
117	58				
118	57				
119	52				
120	55				
121	52				
122	55				
123	50				
124	56				
125	69				
126	67				
127	55				
128	51				
129	63				
130	68				
131	65				
132	55				
133	63				
134	58				
135	57				
136	52				
137	55				
138	52				
139	55				
140	50				
141	56				
142	69				
143	67				
144	56				
145	60				
146	80				
147	55				
148	51				
149	63				

Table 1 cont'd

Num.	Healthy control	Lung cancer			
	Age	Age	immunocytochemistry markers		
			CK7	Napsin A	TTF-1
150	68				
151	65				
152	55				
153	63				
154	58				
155	57				
156	52				
157	55				
158	52				
159	55				
160	50				
161	56				
162	69				
163	67				
164	55				
165	51				
166	73				
167	78				
168	75				
169	55				
170	73				
171	58				
172	57				
